# Investigation of antimicrobial susceptibility, class I and II integrons among *Pseudomonas aeruginosa* isolates from hospitalized patients in Isfahan, Iran

**DOI:** 10.1186/s13104-018-3901-9

**Published:** 2018-11-12

**Authors:** Jamshid Faghri, Samereh Nouri, Saba Jalalifar, Mehrdad Zalipoor, Mehrdad Halaji

**Affiliations:** 1 Department of Microbiology, School of Medicine, Isfahan University of Medical Sciences, Isfahan, Iran; 2 Department of Microbiology, Clinical Laboratory of ALZAHRA Medical Center, Isfahan, Iran; 3 Students Research Committee, Isfahan University of Medical Sciences, Hezar Jarib St, Isfahan, Iran

**Keywords:** *Pseudomonas aeruginosa*, Antibiotic resistance, Integrons

## Abstract

**Objectives:**

The role of integrons in the transfer of antibiotic resistance is one of the important issues, therefore, this study is aimed to investigate antibiotic resistance pattern and prevalence of class 1 and 2 integrons in *P. aeruginosa* isolated.

**Results:**

Out of 72 confirmed *P. aeruginosa* isolates, 50% were from ICU patients. Antibacterial susceptibility pattern showed that isolates were most resistant to ceftazidime (76.4%) and colistin was the most effective antibiotic (100%) and molecular analysis of class I and II integrons showed 55.5% and 29.1% of isolates were positive, respectively and the proportions of MDR isolates were significantly higher among integron-positive isolates with 73.6% compared to negative isolates with 22.9%. Our results showed that there was a correlation among class 1 and 2 integrons with MDR *P. aeruginosa* isolates. According to the importance of integrons in acquisition and dissemination of antibiotics resistance genes, the performance of antibiotic surveillance programs and investigating the role of integrons is recommended to control the spreading of antibiotics resistance genes.

## Introduction

*Pseudomonas aeruginosa* is a non-fermenting aerobic Gram-negative bacillus known as a significant pathogen leading to severe infections in hospitals and rarely causes infection in the natural host [1]. *P. aeruginosa* is an important opportunistic pathogen leading to severe infections in patients with cystic fibrosis, neutropenia, iatrogenic immunosuppression, or severe burns [2, 3]. The increasing use of antibiotics is likely the main reason for the evolution of multidrug-resistant (MDR) *P. aeruginosa* outbreaks in clinical settings [4, 5]. Generally, bacterial resistance can be explained by either the mutation of genes or exchangeable genetic elements such as plasmids, transposons, and integrons [6]. Integrons are transportable genetic elements which can transfer the antibiotic resistance genes. These elements can be placed in different parts of plasmids and chromosomes. Integrons are able to capture external drug resistance gene cassettes and incorporate them using site-specific recombination. It is potentially a major agent in the rapid spread of resistance in bacteria, especially among the Gram-negative [6]. The overall structure of integrons includes an integrase gene, two stable conservative reigns called sul1 and int1 as well as a variable region of gene cassettes between the two conserved segments [7]. According to the integrase gene, four classes of integrons have been described in Gram-negative organisms, however, class 1 integrons have been described to be the most prevalent in clinical isolates, carrying single or several gene cassettes, which confer resistance to a wide range of antibiotics such as β-lactams, fluoroquinolones, aminoglycosides, chloramphenicol, erythromycin, antiseptics, and disinfectants [7]. The second class of integrons (class 2) is commonly found to be associated with the Tn7 transposon family and it contains integrase gene (intI2), one recombinant location (aatI 2) and a cassette gene without SulI in its 3′ terminal region. Class 2 integrons have been reported in some species of Gram-negative bacteria such as Acinetobacter, *Enterobacteriaceae*, Salmonella and Pseudomonas [8]. Compared with class 2 integron, class 3 integron contains a similar structure and this class of integrin have been described as rare and found only within a few microorganisms such as *Serratia marcescens*, *Klebsiella pneumoniae* and *P. aeruginosa* [8–10]. Furthermore, class 4 integron which harbor a wide range of gene cassettes encoding antibiotic resistance and pathogenicity was firstly identified on the small chromosome of *Vibrio cholerae* [8].

Regarding the significant role of *P. aeruginosa* in nosocomial infections and also the role of integrons in the dissemination of antibiotic resistance, this study is aimed to evaluate the frequency of class 1 and 2 integrons in *P*. *aeruginosa* isolates of nosocomial infection in order to determine the antibiotic resistance pattern of this bacterium.

## Main text

### Methods

A cross-sectional study was performed during the period April 2016 to August 2016 at a teaching hospital affiliated to Isfahan University of Medical Sciences, Isfahan, Iran. The non-replicated *P. aeruginosa* isolates were obtained from various clinical specimens such as blood, wounds, urine and other samples and transported to the laboratory for further analysis. The specimens were cultured on blood agar and MacConkey’s agar (Merck, Germany) and incubated overnight at 37 °C. Bacterial isolates were identified as *P. aeruginosa* using standard microbiological methods, including Gram staining, production of acid from different sugars, catalase, oxidase, motility, and genotypic method (the presence of the *tox*-*A* gene). The confirmed isolates were stored at − 80 °C in brain heart infusion (BHI) broth containing 20% glycerol [11, 12].

Antibiotic susceptibility was performed based on disk diffusion method on Mueller–Hinton agar (Himedia, India) according to the Clinical and Laboratory Standards Institute (CLSI) recommendation for meropenem (10 μg), cefepime (30 μg), ceftazidime (30 μg), piperacillin–tazobactam (100/10 μg), colistin–sulfate (10 μg), amikacin (30 μg), ciprofloxacin (5 μg) and levofloxacin (μg); disks (MAST, Merseyside, UK). *P. aeruginosa* ATCC 27853 was also tested as the control positive. However, multi-drug resistant isolates were defined as those resistant to at least three of antibiotics classes [13].

A simple boiling method was used to extract genomic DNA from *P. aeruginosa* isolates as described previously [14]. The polymerase chain reaction (PCR) was carried out to detect the *tox*-*A* gene and the presence of class 1 and 2 integrons cassette using the specific primers [15].

PCR amplification consisted of a pre-denaturation step at 94 °C for 5 min, followed by 35 cycles of denaturation at 94 °C for 45 s, primer annealing at 54 °C for *intI1* and *intI2* and extension at 72 °C for 60 s, and a final extension at 72 °C for 7 min. The PCR products were analyzed on agarose gel with KBC power load dye (CinnaGen Co. Iran) and finally visualized in gel documentation system. Positive results were confirmed by direct sequencing of the PCR products.

SPSS Statistics (IBM Corp. Released 2012. IBM SPSS Statistics for Windows, Version 21.0. Armonk, NY: IBM Corp.) was used for statistical analysis. Chi square or Fisher’s exact test was used to determine any statistical association. Statistical significance was regarded as *p*-values < 0.05. The results are showed using descriptive statistics in terms of relative frequency.

### Results

A total of 72 confirmed *P. aeruginosa* isolates were obtained from various clinical samples of a selected teaching hospital in Isfahan, Iran. Totally, 66.7% (48/72) *P. aeruginosa* isolates were obtained from male and 33.3% (24/72) from female hospitalized patients. The distribution of *P. aeruginosa* isolates in different hospital wards and clinical specimens showed that half of the isolates were collected from ICU and most of the isolates were isolated from trachea samples. Table 1 shows the distribution of clinical specimens and different wards based on integron-positive *P. aeruginosa* isolates.

**Table 1:** Frequency of integron-positive *P. aeruginosa* isolates based on clinical specimens and different wards

Wards	Integron-1 positive no. (%) = 40	Integron-2 positive no. (%) = 21	Total no. = 61
ICU	23 (57.5)	11 (52.4)	34
Emergency	7 (17.5)	4 (19)	11
Surgery	5 (12.5)	2 (9.5)	7
Internal medicine	3 (7.5)	1 (4.8)	4
Others	2 (5)	3 (14.3)	5

Clinical sample	Integron-1 positive no. (%)	Integron-2 positive no. (%)	Total no. = 61
Trachea	18 (45)	9 (43)	27
Urine	9 (22.5)	5 (23.7)	14
CSF	2 (5)	–	2
Wound	5 (12.5)	3 (14.3)	8
Others	6 (15)	4 (19)	10
MDR	32/40	15/21	53 (73.5)

Antibacterial susceptibility pattern showed a high rate of antibiotic resistance to ceftazidime 76.4% followed by ciprofloxacin 73.6%. However, all of the isolates were susceptible to colistin (100%) and the lowest antibiotic resistance rates were seen against amikacin (41.6%) followed by piperacillin/tazobactam (48.6%).

PCR amplification of the two class 1 and 2 integrons cassette showed that 55.5% and 29.1% of isolates were positive for class 1 and 2 integrons, respectively.

The high level of resistance in class 1 and 2 integron-positive isolates were seen to ceftazidime (82.5) and ciprofloxacin (76.2%), respectively, whereas amikacin and piperacillin/tazobactam were the most effective antibiotics to integron-positive isolates. The full antibiotic resistance pattern of the integron-positive and integron-negative isolates are presented in Tables 2 and 3. According to our results, a significant correlation between the presences of class I integron and a higher rate of resistance related to meropenem was seen (*p*-value < 0.001). The rate of MDR phenotype among integron-positive isolates (73.6%; n = 53) were significantly higher in compared to integron-negative isolates with 22.9% (n = 16).

**Table 2:** Antibiotic susceptibility pattern of class 1 integron-positive and integron-negative of *P. aeruginosa* strains

Antibiotics	Integron-1 positive n = 40 No. (%)	Integron-1 negative n = 32 No. (%)	*p*-value
Meropenem	30 (75)	13 (40.5)	0.003
Cefepime	31 (77.5)	21 (65.6)	0.2
Ceftazidime	33 (82.5)	22 (69)	0.17
Piperacillin/tazobactam	23 (57.5)	12 (37.5)	0.09
Ciprofloxacin	31 (77.5)	22 (69)	0.4
Levofloxacin	30 (75)	21 (65.6)	0.38
Amikacin	19 (47.5)	11 (34.4)	0.36
Colistin	0	0	–

**Table 3:** Antibiotic susceptibility pattern of class 2 integron-positive and integron-negative of *P. aeruginosa* strains

Antibiotics	Integron-1 positive n = 40 No. (%)	Integron-1 negative n = 32 No. (%)	*p*-value
Meropenem	30 (75)	13 (40.5)	0.003
Cefepime	31 (77.5)	21 (65.6)	0.2
Ceftazidime	33 (82.5)	22 (69)	0.17
Piperacillin/tazobactam	23 (57.5)	12 (37.5)	0.09
Ciprofloxacin	31 (77.5)	22 (69)	0.4
Levofloxacin	30 (75)	21 (65.6)	0.38
Amikacin	19 (47.5)	11 (34.4)	0.36
Colistin	0	0	–

### Discussion

*Pseudomonas aeruginosa* is one of the most important pathogen causing a wide range of infections in the hospitals and healthcare settings [16]. The spread of resistance to antimicrobial agents and the emergence of clinical multidrug resistance *P. aeruginosa* isolates in clinical settings has become a serious problem in the treatment of nosocomial infections worldwide [17, 18]. Resistance to antimicrobial agents can be originated from many resistance genes that present as gene cassettes and transferred through integrons which are located on transmissible plasmids, the chromosome, and transposons [10]. In the present study, based on antibiotic susceptibility pattern, colistin was the most effective antibiotic agent with the susceptibility rate of 100%, while most of *P. aeruginosa* isolates showed high resistance rate to antimicrobial agents. In accordance to our results, Khosravi et al. from south of Iran, Mobaraki et al. and Goli et al. from Northwest of Iran, reported that colistin was the most effective and the drug of choice in the management of nosocomial infection [19–21].

In another study carried out by Fazeli et al. [22] in our region, lower resistance rate was described for quinolones and cephems agents. Among them, a resistance rate of 63% and 63.1% were reported for both ciprofloxacin and ceftazidime, respectively. Whereas, our results showed a higher resistance to quinolones and cephems groups especially ciprofloxacin and ceftazidime which demonstrated an increasing trend of this resistance in hospitalized patients in Isfahan. In our finding, the rate of resistance to amikacin was 41% that was lower than reports from the south of Iran and were also reported in other parts of the world [19, 21–24]. To our knowledge, this is the first study addressing the investigation of a different class of integron in *P. aeruginosa* isolates in our region, therefore, this present study demonstrated the importance and frequency of classes 1 and 2 integrons and the importance of them in relation to high antibiotic resistance *P. aeruginosa* isolates.

In the present study, 55.5% and 29% of *P. aeruginosa* isolates were contained class 1 and 2 integrons, meanwhile, 16.6% of isolates carried both classes of integron genes, simultaneously.

The overall data show that the prevalence of *int1* gene in clinical isolates was closest to most of the previous investigations from Iranian studies ranging from 27.5 to 66% and the results of studies conducted in other countries [20, 21, 25–28].

The emergence of the high frequency of *intI1* in our region may explain a serious concern in the future and can cause an increase and dissemination of antibiotic resistance that is related to the presence of class 1 integron. In similar studies from Iran, due to inappropriate use of antibiotics, the origin of infections and geographical distribution, the prevalence of class 2 gene in clinical isolates of *P. aeruginosa* is variable. Accordingly, in contrast to our finding, other studies have reported a variable frequency of class 2 integrons among clinical *P. aeruginosa.* Khosravi et al., Hosseini Pour et al. from Ahvaz city and Mobaraki et al. from Northwestern Iran showed 0%, 52% and 25.5% isolates harboring class 2 integrons, respectively [19, 21, 29].

According to the previous finding, there is an association between the presence of multiple resistance and different classes of Integron, especially class 1 integrons [30]. Bearing in mind our results, in compared to negative isolates, the proportion of MDR isolates among integron-positive isolates were considerably higher, which can confirm the importance of these elements in the spreading of resistance genes among pathogens.

In agreement with our findings, a high rate of MDR isolates among integron-positive were reported by Ebrahimpour et al. [31]. Moreover, Khosravi et al. [19] also showed there is a correlation between the presence of class 1 integrons and MDR isolate and high antimicrobial resistance especially for gentamicin (94.62%), ciprofloxacin (93.54%), and meropenem (90.32%).

Although the resistance rate of meropenem was significantly differenced in class 1 integron-positive than in integron negative isolates, there was no significant correlation between most antibiotics used and integron-positive isolates in our study. Therefore, the use of inappropriate antibiotics, selective pressure, and other factors may be considered for high resistance to antibiotics. The results of our finding describe the importance of class 1 and 2 integrons in multiple antibiotic resistance and its association with MDR *P. aeruginosa* isolates. Therefore, integrons play an important role in acquisition and dissemination of antibiotics resistance genes among these pathogens, so, management of infection control policies and the appropriate use of antibiotics is necessary for control the spreading of antibiotics resistance genes.

## Limitations

The lack of investigation and characterization of resistance gene cassettes associated with class 1 and 2 integrons can be mentioned as one of the main limitations of the present study.

## Abbreviations

aatI 2: recombinant location
BHI: brain heart infusion
CLSI: Clinical and Laboratory Standards Institute
intI2: integrase gene
PCR: polymerase chain reaction
MDR: multidrug-resistant
