# Correction to: Investigation of antimicrobial susceptibility, class I and II integrons among *Pseudomonas aeruginosa* isolates from hospitalized patients in Isfahan, Iran

**DOI:** 10.1186/s13104-019-4122-6

**Published:** 2019-02-12

**Authors:** Jamshid Faghri, Samereh Nouri, Saba Jalalifar, Mehrdad Zalipoor, Mehrdad Halaji

**Affiliations:** 1 Department of Microbiology, School of Medicine, Isfahan University of Medical Sciences, Isfahan, Iran; 2 Department of Microbiology, Clinical Laboratory of ALZAHRA Medical Center, Isfahan, Iran; 3 Students Research Committee, Isfahan University of Medical Sciences, Hezar Jarib St, Isfahan, Iran

## Correction to: BMC Res Notes (2018) 11:806 10.1186/s13104-018-3901-9

Following publication of the original article [1], an error was reported in the tables due to a typesetting mistake. Table 3 is presented as a duplicate of Table 2. In this Correction, the correct presentation of Table 3 is shown.

**Table 3:** Antibiotic susceptibility pattern of class 2 integron-positive and integron-negative of *P. aeruginosa* strains

Antibiotics	Integron-2 positive n = 21 No. (%)	Integron-2 negative n = 51 No. (%)	*p*-value
Meropenem	14 (66.6)	29 (57)	0.4
Cefepime	15 (71.4)	37 (72.5)	0.9
Ceftazidime	15 (71.4)	40 (78.5)	0.5
Piperacillin/tazobactam	10 (47.6)	25 (49)	0.9
Ciprofloxacin	16 (76.2)	37 (72.5)	0.7
Levofloxacine	14 (66.6)	37 (72.5)	0.6
Amikacin	11 (52.4)	19 (37.2)	0.3
Colistin	0	0	–

The publisher apologises to the authors and readers for the inconvenience.
